# Comparison of predictive models in postoperative nausea and vomiting in patients undergoing breast cancer surgery

**DOI:** 10.1007/s00520-024-08781-z

**Published:** 2024-08-08

**Authors:** Gülseren Maraş, Halil Kalaycı, Özlem Ceyhan

**Affiliations:** 1 Erciyes University, Faculty of Health Sciences, Surgery Nursing, Kayseri, Türkiye; 2 Erciyes University, Faculty of Health Sciences, Internal Medicine Nursing, Kayseri, Türkiye

**Keywords:** Apfel score, Breast cancer surgery, Koivuranta score, Nausea-vomiting, Risk factors

## Abstract

**Background:**

Post-operative nausea and vomiting remain an unresolved concern in Türkiye and some parts of the world, impacting the quality of the patient's recovery process and diminishing overall satisfaction.

**Objective:**

This study was conducted as a descriptive investigation to compare the incidence of nausea and vomiting following breast cancer surgery with the nausea and vomiting risk scores proposed by Apfel and Koivuranta.

**Methods:**

This study was conducted with 100 patients admitted to the General Surgery service of a university hospital between 31 August 2019 and 31 May 2021 for breast cancer surgery. The patient information form developed by the researchers, Apfel Nausea and Vomiting Risk Score, and Koivuranta Nausea and Vomiting Risk Score were used as data collection tools.

**Results:**

It was identified that 61% of the patients experienced nausea and vomiting within the initial 24 h following surgery. A significant correlation was found between age, post-operative opioid use, motion sickness or history of PONV, and nausea and vomiting (*p* < 0,05). The sensitivity of the Apfel score obtained was 80%, the specificity was 46%, and the AUC value was 0.686. The sensitivity of the Koivuranta score was 80%, the specificity was 35%, and the AUC value was 0.675 (*p* < 0.05).

**Conclusion:**

It has been observed that patients experience high rates of nausea and vomiting after breast cancer surgery and that the Apfel and Koivuranta Risk Scores are equally applicable in predicting post-operative nausea and vomiting.

## Introduction

Breast cancer ranks first among the most common cancer types occurring worldwide, with an incidence of 11.7%. Breast cancer is the second most common cancer in Türkiye and the first among the cancer types seen in women, with an incidence rate of 10.3% (24,175) [1]. Anesthesia used during the surgical treatment of breast cancer can cause problems such as nausea-vomiting, pain, constipation, diarrhea, edema, urinary retention, loss of appetite, stress, fear, anxiety, and depressed mood [2]. Post-operative nausea and vomiting (PONV) are the most common causes of post-operative dissatisfaction. PONV is nausea, retching, or vomiting within 24–48 h after surgery [3]. A widespread problem of patients after surgery and anesthesia, it is seen in 20–30% of all surgical patients and 70–80% of high-risk patients worldwide [4]. The incidence of nausea and vomiting after breast cancer surgery has been reported as high as 30–75% in the literature [4–8]. It is predicted that 4,07,871 women will be diagnosed with breast cancer by 2040 and have surgery to treat it. As a result, post-operative nausea and vomiting may be a significant clinical problem [1].

Considering the complications caused by PONV, which can develop due to surgery and anesthesia, risk factors should be defined to prevent them with prophylactic regimens and optimize their treatment. PONV has numerous risk factors associated with the patient, surgery, and anesthetic. The most vital predictive factors for PONV were female gender, motion sickness or history of PONV, non-smoking, and intra- and post-operative opioid use. Among these factors, the female gender outweighs the risk factor. As breast surgery related to breast cancer is carried out almost entirely in women, the severity of the issue increases [3, 9].

PONV can cause dehydration, bleeding, wound dehiscence, and aspiration, resulting in decreased patient comfort and quality of life, more medical expenses due to electrolyte imbalance, and prolonged hospital stay in long-term cases [4]. For this reason, the evidence-based interventions used in treating PONV emphasize integrating guidelines into practice for the prevention and management of PONV [10]. Apfel and Koivuranta predictive models are recommended among the applications used to identify and evaluate PONV risk factors and manage perioperative risks [11, 12].

Although PONV is a condition that affects the quality of the patient's recovery process, reduces patient satisfaction, and increases the potential for rehospitalization in high-risk patients, it is still not a subject that has been studied much [9]. This descriptive study was conducted to determine the prevalence of nausea-vomiting and to compare the predictors of Apfel and Koivuranta nausea-vomiting risk scores after breast cancer surgery.

## Method

### Design

The study was conducted with women who underwent breast cancer surgery in the general surgery service of a university hospital in Türkiye. In the clinic where the study was conducted, there is no protocol for predicting and preventing post-operative nausea and vomiting in patients. Specifically, there is an absence of a structured protocol delineating the risk stratification for nausea and vomiting and the subsequent administration of pertinent pharmacological interventions. Antiemetics are not routinely administered in the operating room and clinic. Antiemetic drugs are administered in the clinic when post-operative nausea and vomiting are observed. Dopamine receptor antagonists (Metoclopromide) are administered when patients complain of nausea, and serotonin receptor antagonists (Ondansetron, Granisetron, Dolasetron) are administered to patients who begin vomiting.

### Sampling

According to the information obtained from the 2018–2019 university hospital data, the study population consists of 100 female patients hospitalized in the general surgery service and undergoing surgery for breast cancer. The sample of the study was calculated based on the frequency of post-operative nausea and vomiting, which Wesmiller et al. reported as 29.8% in their study on nausea and vomiting after breast cancer surgery [5]. In light of this information, post-operative nausea and vomiting frequency was determined as the expected incidence of 30%. The minimum number of patients required was defined as 77 with the formula for determining the sample size at a 95% confidence interval. No sample selection was made, and all patients who agreed to participate in the study and signed the informed consent form were included in the sample, and the entire population (100 people) was reached. Inclusion criteria in the study: 18 years or older, female patients diagnosed with breast cancer, planned for elective breast surgery, no communication problems, no known psychiatric disorder, and patients who volunteered for the study.

### Data collection forms

The patient information form developed by the researchers, [2, 4, 9, 11, 12], Apfel Nausea and Vomiting Risk Score, and Koivuranta Nausea and Vomiting Risk Score were used as data collection tools. The patient information form consists of 25 questions examining sociodemographic (gender, education level, marital status, place of residence, BMI, etc.) and clinical characteristics (chronic disease, motion sickness, chemotherapy status, medications used, surgical history, anesthetic type, duration of surgery, post-operative use of antiemetic agents, pain, nausea, and vomiting, etc.). The Apfel Risk Score consists of four items, and the Koivuranta Risk Score consists of five items. Patients were asked whether they experienced nausea after surgery, and the severity of nausea in patients who experienced nausea was determined with the Visual Analog Scale. Vomiting was measured by counting.

### Apfel and Koivuranta risk scores

The simplified risk score developed by Apfel et al. [11] for adults includes four independent risk factors. According to this scoring, the risk factors are female gender, non-smoking, history of post-operative nausea, vomiting (PONV) or motion sickness, and post-operative opioid use. The incidence of none of these risk factors is 10%, only one is 21%, two is 39%, three is 61%, and four are 79%.

Koivuranta et al. [12] is a risk score calculation with five variables. According to this scoring, the risk factors are female gender, non-smoking, history of post-operative nausea and/or vomiting (PONV), motion sickness, and prolonged operation time (> 60 min). One of these risk factors is 17%, two risk factors are 18%, three risk factors are 42%, four 74%, and five 87%.

### Visual analog scale

The study measured the patients' pain and nausea severity using the Visual Analogue Scale (VAS) scale of Boogaerts et al. (2000), graded between 0 and 10 cm. VAS was created using a 10 cm-long vertical line. The left end corresponds to “0” (no symptom)’ and the right to “10” (unbearable symptom). The patient was asked to mark the place on this line indicating his/her condition. In the study, the severity of pain and nausea of the patient was classified as 0 (no nausea-vomiting/no pain), 1–3 (mild), 4–6 (moderate), and 7–10 (severe) [13].

### Data collection

Researchers visited patients' rooms before surgery, evaluated risk scores, and administered patient data collection forms. They recorded how many times the patients needed antiemetics 24 h after the surgery and how many times they experienced nausea and vomiting in the 24 h after the surgery. In patients experiencing nausea, the severity was measured with the VAS, and vomiting was measured with the count.

#### Post-operative nausea

Nausea is an uncomfortable sensation accompanied by relaxation of the gastrointestinal tract, duodenal peristalsis, and vegetative symptoms [14].

#### Post-operative vomiting

Within 24 h after the surgery, the patient experiences one or more periods of undigested food in the stomach expelled through the mouth [15].

### Ethical considerations

Academic committee permission (2019/04) and ethical approval from the Erciyes University Clinical Research Ethics Committee (2019/598) were obtained to conduct the research. A written informed consent form was obtained from the patients who volunteered to participate in the study.

### Analyses

SPSS 25.0 (IBM SPSS Statistics Standard Concurrent User ver. 25) program was used for statistical data analysis. Descriptive statistics are the number of units (n) and percentage (%). Correlation statistics were made using the Fisher and Pearson chi-square tests and logistic regression analysis. Receiver Operating Characteristic (ROC) Analysis was used to evaluate the sensitivity and specificity of risk scores. The data were assessed at the 95% confidence interval and the significance level of *p* < 0.05.

## Results

The mean age of the participants, all women, was 51.51 ± 13.71. It was determined that endotracheal intubation was applied to 82% of the patients, the operations of 52% took 121–240 min, and 97% had post-operative pain. Although not specified in the Table, mastectomy was performed in half (50.0%) of the patients, and Breast Conserving Surgery (BCS) was performed in the other half (50.0%). Nausea was observed in 61% of the patients, and vomiting was observed in 20%. Moderate pain was observed in 51% of the patients, and severe nausea was observed in 25%. Although it is not stated in the Table, it was determined that the average nausea severity of the patients who were administered antiemetics was 6.25 out of 10. The average nausea severity of those not administered antiemetics was 2.87 out of 10. Considering the distribution of nausea and vomiting risk factors, it was determined that 87.0% had an operation more extended than 60 min, 83.0% had vehicle sensitivity, 79.0% were administered opioids postoperatively, 69.0% were non-smokers, 69.0% had a history of PONV. According to the Apfel Risk score, the distribution of the patients from 1 to 4 points was 8%, 22%, 53%, and 17%, and the distribution from 1 to 5 points according to the Koivuranta Risk score was 3%, 23%, 46%, 23%, and 5% respectively (Table 1).

**Table 1:** Descriptive characteristics of breast surgery patients

	*n* (%)
Anesthesia airway vehicle	
Endotracheal Intubation	82 (82)
Laryngeal Mask (LMA)	18 (18)
Surgery duration	
< 60 min	13 (13)
60- 120 min	25 (25)
121–240 min	52 (52)
> 240 min	10 (10)
Postoperative pain	
Yes	97 (97)
No	3 (3)
Postoperative nausea	
Yes	61 (61)
No	39 (39)
Postoperative vomiting	
Yes	20 (20)
No	80 (80)
Pain severity	
None	3 (3)
Mild (1–3)	18 (18)
Moderate (4–6)	51 (51)
Severe (7–10)	28 (28)
Nausea severity	
None (0)	39 (39)
Mild (1–3)	13 (13)
Moderate (4–6)	23 (23)
Severe (7–10)	25 (25)
Apfel risk factors	
Female Gender	100 (100)
History of PONV or Motion Sickness	42 (42)
No Smoking	69 (69)
Postoperative Opioid Use	79 (79)
Koivuranta risk factors	
Female Gender	100 (100)
Motion Sickness	17 (17)
No Smoking	69 (69)
History of PONV	31 (31)
Surgical Duration > 60 min	87 (87)
Apfel risk scores	
1 Risk Score	8 (8)
2 Risk Score	22 (22)
3 Risk Score	53 (53)
4 Risk Score	17 (17)
Koivuranta risk scores	
1 Risk Score	3 (3)
2 Risk Score	23 (23)
3 Risk Score	46 (46)
4 Risk Score	23 (23)
5 Risk Score	5 (5)
Total	100 (100)

*PONV* Postoperative nausea and vomiting

**Fig. 1:**
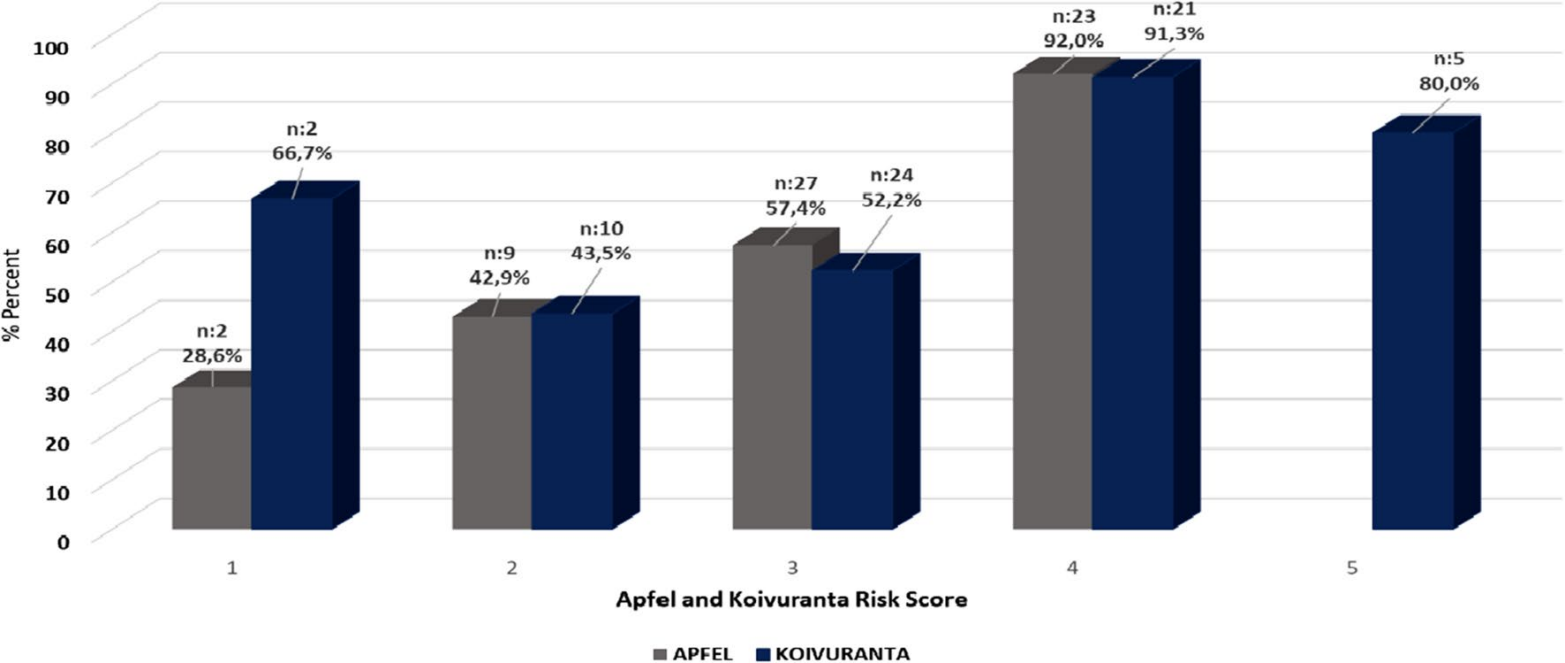
Apfel and Koivuranta risk score and nausea-vomiting rates

Post-operative nausea and vomiting are given in Fig. 1 and Table 2 according to Apfel and Koivuranta risk scores: 28.6% of patients with one risk factor, 42.9% with two risk factors, 57.4% with three risk factors, and 92.0% with four risk factors had nausea-vomiting. According to the Koivuranta nausea and vomiting risk score, 66.70% of patients with one risk factor, 43.5% in patients with two risk factors, 52.20% in patients with three risk factors, 91.3% of patients with four risk factors, and 80% of patients with five risk factors had nausea-vomiting.

**Table 2:** Presence of nausea and vomiting by risk score and other factors

	Nausea and Vomiting		Test value
	Yes	No	Chi square (*X*^*2*^)
	*n* (%)	*n* (%)	
Apfel risk score			
1	2 (28.6)	5 (71.4)	0.000*
2	9 (42.9)	12 (57.1)	
3	27 (57.4)	18 (42.6)	
4	23 (92.0)	2 (8.0)	
Koivuranta risk score			
1	2 (66.7)	1 (33.03)	0.002*
2	10 (43.5)	13 (56.5)	
3	24 (52.2)	22 (47.8)	
4	21 (91.3)	2 (8.7)	
5	4 (80.0)	1 (20.0)	
Age			
22–42	20 (76.9)	6 (23.1)	0.006**
43–51	15 (57.7)	11 (42.3)	
52–60	19 (73.1)	7 (26.9)	
61–90	7 (31.8)	15 (68.8)	
Postoperative opioid use			
Yes	55 (48.2)	24 (30.8)	0.001**
No	6 (28.6)	15 (71.4)	
Motion sickness			
Yes	14 (82.4)	3 (17.6)	0.048**
No	47 (46.2)	36 (43.4)	
History of PONV			
Yes	22 (71.0)	9 (29.0)	0.171**
No	39 (56.5)	30 (43.5)	
Postoperative opioid use or history of PONV			
Yes	31(73.8)	11(26.2)	0.025**
No	30 (51.7)	28 (48.3)	
Surgical duration			
≤ 60 Minutes	6 (46.2)	7 (53.8)	0.239**
> 60 Minutes	55 (62.2)	32 (33.9)	

*PONV* Postoperative nausea and vomiting

*Fisher’s, **Pearson Chi-Square

*p* < .05

According to Table 2, it was determined that there was a statistically significant difference between post-operative nausea and vomiting risk factors such as age, post-operative opioid use, motion sickness, and motion sickness, or history of PONV and nausea and vomiting (*p* < 0.05). There was no statistically significant difference between chronic disease, anesthesia airway device, operation time, pain, and pain intensity (*p* > 0.05).

In Table 3, the risk factors that are predictors of nausea and vomiting are analyzed using the Logistic Regression (binary) model. The test determined that the Apfel risk score explained 21.9% of nausea and vomiting, and the Kovurianta risk score explained 12.5% (*p* < 0.05).

**Table 3:** Logistic regression model of risk factors associated with postoperative nausea and vomiting

Independent variable	B†	SE†	Exp(B)	*p*	95% CI†	Nagelkerke R^2^	Model *p*
Apfel risk factors							
Motion Sickness or History of PONV	1.013	0.706	2.753	0.152	0.689 to 10.993	0.219	**0.029**
Postoperative Opioid Use	1.572	0.551	4.818	**0.004**	1.636 to 14.189		
No Smoking	0.619	0.472	1.856	0.190	0.736 to 4.683		
Koivuranta risk factors							
Motion Sickness	1.250	0.693	3.491	0.071	0.898 to 13.565	0.125	**0.029**
No Smoking	0.735	0.456	2.085	0.107	0.854 to 5.095		
History of PONV	-0.565	0.484	0.569	0.243	0.220 to 1.467		
Surgical Duration > 60 min	-0.649	0.626	0.523	0.523	0.153 to 1.782		

*PONV* Postoperative nausea and vomiting, *†B* Unstandardized regression coefficient, *SE* Standard error; *β* Standardized regression coefficient; *CI* Confidence interval

*p* < .05

**Fig. 2:**
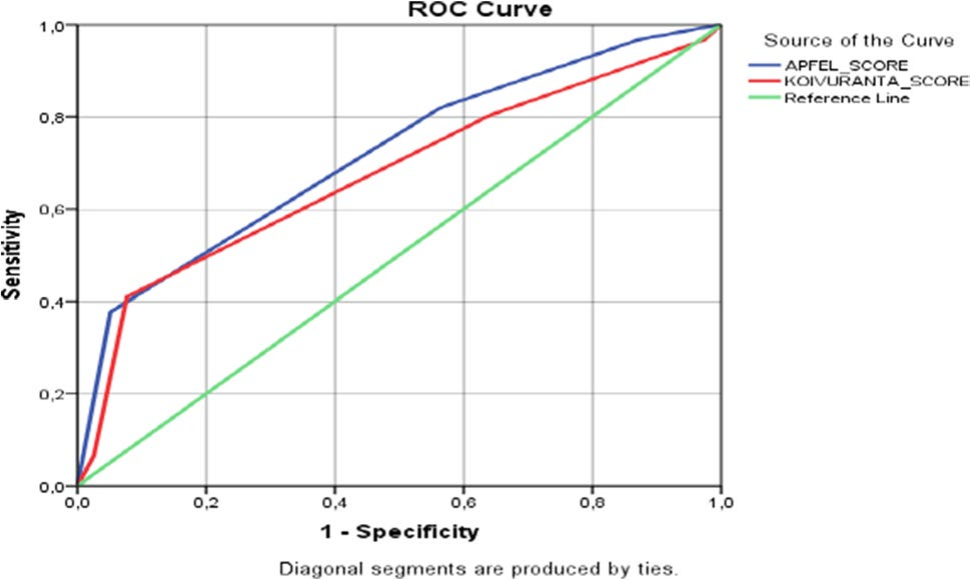
Apfel and Koivuranta ROC analysis

The sensitivity of the Apfel risk score obtained from the study was 82.0%, the specificity was 56.4%, the cut-off value was 2.5, and the AUC value was 0.718 (0.618–0.817). The sensitivity of Kovurianta risk scoring was 80.3%, the specificity was 64%, the cut-off value was 2.5, and the AUC value was 0.675 (0.570–0.871) (*p* < 0.05) (Fig. 2).

## Discussion

Post-operative nausea and vomiting continue to be significant problems that negatively affect post-operative outcomes. Guidelines based on risk prediction models are not adequately implemented, and PONV cases cannot be managed reliably. Appropriately designed studies are needed to incorporate risk prediction models into PONV management protocols [16]. Prevention and management of post-operative nausea and vomiting require multidisciplinary teamwork and pharmacological and non-pharmacological interventions. Nurses are the healthcare professionals who interact most frequently and most closely with patients and their families. Therefore, they play a vital role in PONV management and prevention. Nurses' responsibilities include assessing risk factors for nausea and vomiting [17]. Integrating routine risk stratification tools into the clinic to determine the prevalence of PONV in specific surgeries, such as breast cancer surgery, may help surgeons, nurses, and patients better manage their disease.

In this study, the relationship between the occurrence of post-operative nausea and vomiting and risk factors included in the scoring criteria, such as age, female gender, motion sickness or history of PONV, non-smoking status, post-operative opioid use, and duration of surgery, has been investigated. Nausea was observed in 61% of the patients, and vomiting in 20%. Moderate pain was observed in 51% of the patients, and severe nausea was observed in 25%. It was determined that the average nausea severity of the patients who were administered antiemetics was 6.25 out of 10, and the average nausea severity of those who were not administered antiemetics was 2.87 out of 10. This shows that although antiemetics are effective in vomiting, they are ineffective in nausea and nausea severity. This may be related to the consequences of not applying antiemetic prophylaxis and multimodal management strategies to patients. An incidence of PONV reaching 30–68% in the first 24 h after the surgery has been reported in female patients undergoing breast and gynecological surgery, despite prophylactic antiemetic treatment. Although published data are limited, it is emphasized that other variables such as pre-and post-menopausal sex hormone levels (especially estrogens), preoperative psychosocial status, pharmacogenomics, polymorphism, and ethnicity should be considered as independent risk factors in female patients at elevated risk [18].

Studies in the literature have reported that the incidence of PONV after breast cancer surgery varies between 35–75% [2, 8, 9, 15–17]. A significant correlation was found between age, post-operative opioid use, motion sickness or history of PONV, and nausea and vomiting (*p* < 0, 05). It is also seen in the literature that nausea and vomiting are significantly higher in patients of the female gender, age, non-smoking, motion sickness or history of PONV, opioid use, and BMI [17–21].

In the study by Apfel et al. [11], the frequency of nausea and vomiting is evaluated between 0 and 4. The scoring system predicts an incidence of nausea and vomiting of 10% for 1 risk factor, 40% for 2 risk scores, 60% for 3 risk scores, and 80% for 4 risk scores. Choy et al. determined that the PONV rate was 10%, 22%, 26%, and 60% for those with a risk score of 1, 2, 3, and 4, respectively [22]. Similarly, this study determined that nausea and vomiting developed in 28.6% for 1 score, 42.9% for 2 scores, 57.4 for 3 scores, and 92.0% for 4 scores. The study is similar to the literature.

Koivuranta et al. [12] studied a five-point score in the Koivuranta risk scoring. In return for scores ranging from 1–5, nausea was 18%, 42%, 54%, 74%, 87%, and vomiting was 7%, 17%, 25%, 38%, and 61%, respectively. In the study of Geçit and Özbayır [17], the incidence of nausea and vomiting for Koivuranta risk scores between 1–5 was found to be 18.6%, 37.1%, 26.9%, 12.4%, 2.5%, respectively. In this study, the incidence of nausea and vomiting in patients with nausea and vomiting according to Koivuranta risk scores was found to be 66.70% for score 1, 43.50% for score 2, 52.2% for score 3, 91.30% for score 4 and 81% for score 5.

Post-operative nausea and vomiting risk scores of Ahmed and Lema [23] were determined as the factors associated with motion sickness or history of PONV and a long surgery time as a result of multiple logistic regression analysis. In the study of Elsaid et al. [24], PONV formation was significantly associated only with patients with a history of PONV in the multiple binary logistic regression model. It was stated that the model explained 28.67%. This study determined that the Apfel risk score, the predictor of nausea and vomiting, explained 21.9%, and the Kovurianta risk score explained 12.5%. PONV occurrence was significantly associated only with patients with post-operative opioid use. The study is not like the literature in this aspect.

In the ROC analysis performed to determine the accuracy rate of Apfel risk scoring in predicting patients with and without nausea, the sensitivity of the scoring was 82.0%, the specificity was 56.4%, and the AUC value was 0.718 (0.618–0.817). Kovurianta risk scoring was a sensitivity of 80.3%, a specificity of 64%, and an AUC of 0.675 (0.570–0.871). The size of the area under the ROC curve indicates the statistical significance of the model's discrimination power [25]. This study determined the area under the curve as 0.70-0.80, moderate for Apfel scoring and weak 0.60-0.70 for Kovurianta risk scoring. The Apfel score obtained in the study of Gunawan et al. [26] was reported to have a sensitivity value of 79.5%, a specificity of 45.9%, and an AUC value of 0.701. The sensitivity value of the Koivuranta score is 96.2%, the specificity is 27%, and the AUC value is 0.628. It was concluded that the Apfel score is more accurate and straightforward when estimating PONV. In the study of Wu et al. [27], it was concluded that the predictive power of Apfel (AUC = 0.668, *P* < 0.03) and Koivuranta (AUC = 0.674, *P* < 0.013) predictive models were similar. Two predictive models of Apfel risk score (gender and motion sickness/the history of PONV) were found to explain 12% (two predictive models) of nausea and vomiting. The study is similar to the literature.

## Conclusion

In this study, PONV was observed in most of the patients who underwent breast surgery. Apfel and Koivuranta's nausea and vomiting risk scoring was successful in predicting the risks of nausea and vomiting in patients undergoing breast surgery. The Apfel Nausea and Vomiting Risk Score is more applicable than the Koivuranta Nausea and Vomiting Risk Score in predicting post-operative patient nausea and vomiting. Based on these results, it is recommended that preoperative nausea and vomiting risk scoring be performed effectively and integrated into clinical practices to predict nausea and vomiting and prevent complications. It is also recommended that other independent risk factors such as pre-and post-menopausal sex hormone levels (especially estrogens), preoperative psychosocial status, pharmacogenomics and ethnicity should be considered in female patients at elevated risk.

## Data Availability

No datasets were generated or analysed during the current study.
